# Unveiling the genitourinary phenotype of long COVID: a systematic review and meta-analysis

**DOI:** 10.1007/s11255-026-05073-9

**Published:** 2026-03-08

**Authors:** Daniel Peñaherrera-Vásquez, Alison Reina, Felipe Merlo, Thalía Fajardo-Loaiza, Gabriela Zambrano-Sánchez, Josue Rivadeneira, Luis Fuenmayor-González

**Affiliations:** 1 Unidad de Revisiones Sistemáticas y Metaanálisis-URMA, Facultad de Ciencias Médicas, Universidad Central del Ecuador, 170403 Quito, Ecuador; 2 Carrera de Laboratorio Clínico, Facultad de Ciencias de la Salud, Universidad Técnica de Ambato, 180207 Ambato, Ecuador; 3 Gemeinschaftsklinikum Mittelrhein, 56073 Koblenz, Germany; 4 Hospital de Especialidades Eugenio Espejo, 170403 Quito, Ecuador; 5 Doctorado en Ciencias Médicas, Universidad de la Frontera, 01145 Temuco, Chile; 6 Zero Biomedical Research, 170103 Quito, Ecuador; 7 School of Public Health, Washington University in St. Louis, St. Louis, 63130 USA

**Keywords:** Long COVID, Genitourinary phenotype, Menstrual disorders, Erectile dysfunction, Renal impairment, Systematic review, Meta-analysis

## Abstract

**Importance:**

Long COVID has been associated with persistent multisystemic manifestations. However, genitourinary alterations have not been formally recognized as a distinct phenotype despite growing reports suggesting their relevance for long-term morbidity and quality of life.

**Objectives:**

To determine the frequency and characteristics of genitourinary manifestations in patients with long COVID and to evaluate the evidence supporting the possible emergence of a genitourinary phenotype within long COVID.

**Data sources:**

For this Systematic review and meta-analysis, a comprehensive search was conducted in PubMed (MEDLINE), Scopus, Web of Science, Embase, SciELO, and Bireme-BvS from inception to October 2025, without language or publication date restrictions. Observational studies (cross-sectional, cohort, or case–control) assessing individuals with one or more genitourinary symptoms—such as menstrual alterations, erectile dysfunction, urinary tract symptoms, or renal function decline—persisting ≥ 12 weeks after SARS-CoV-2 infection were included. Studies addressing only acute-phase manifestations, vaccine-related effects, or pre-existing genitourinary conditions were excluded.

**Data extraction and synthesis:**

Data extraction was performed independently by two reviewers following PRISMA guidelines. Risk of bias (RoB) was assessed using the Joanna Briggs Institute checklist for prevalence studies. A random-effects meta-analysis using the Freeman–Tukey double arcsine transformation was applied to estimate pooled proportions, and heterogeneity was quantified using the I^2^ statistic, Cochran’s *Q* test, and the between-study variance (τ^2^).

**Main outcomes and measures:**

The primary outcomes were the pooled frequencies of genitourinary manifestations in long COVID, including menstrual disorders, erectile dysfunction, and renal function decline.

**Results:**

Nine primary studies encompassing 2332 participants from eight countries were included. Most studies (88.9%) presented a low RoB. The pooled frequency of menstrual disorders was 49% (95% CI 24–74), erectile dysfunction 21% (95% CI 16–28), and renal function decline 29% (95% CI 20–39).

**Conclusions and relevance:**

This systematic review and meta-analysis provide evidence supporting the possible emergence of a genitourinary phenotype of long COVID, encompassing menstrual irregularities, erectile dysfunction, cystitis-like symptoms, and renal impairment. Recognition of this potential phenotype is crucial for improving diagnostic accuracy, patient follow-up, and multidisciplinary management. Further high-quality studies are warranted to elucidate the underlying mechanisms and long-term clinical implications.

**Supplementary Information:**

The online version contains supplementary material available at 10.1007/s11255-026-05073-9.

## Introduction

Long COVID is defined by the World Health Organization (WHO) as the persistence of symptoms beyond 12 weeks after acute SARS-CoV-2 infection, in the absence of an alternative explanatory diagnosis. Its global prevalence has been estimated at approximately 10–20% as early as 12 week post-infection. [1]

Although most scientific evidence on long COVID has focused on respiratory, neurological, and cardiovascular manifestations, recent studies suggest that this syndrome does not represent a single clinical entity but rather a heterogeneous group of phenotypes classified by the body systems involved. The first is the cardiovascular and renal phenotype, which includes conditions, such as heart failure, arrhythmias, and kidney disease. The second is the respiratory, sleep, and anxiety phenotype, characterized by respiratory problems, such as chronic cough and dyspnea, along with sleep disturbances and anxiety [2]. The third is the musculoskeletal and nervous system phenotype, marked by musculoskeletal pain and neurological symptoms, such as neuropathy and cognitive dysfunction. Finally, the fourth is the digestive and respiratory phenotype, which involves gastrointestinal symptoms, including nausea and diarrhea, together with respiratory issues [3]. However, a specific genitourinary phenotype has not yet been defined within current long COVID classifications. sSeveral observational studies [4] and case reports have documented genitourinary manifestations, such as long COVID cystitis, erectile dysfunction, menstrual disturbances, overactive bladder symptoms, and even renal function decline [3, 5–7]; nonetheless, this information remains limited and scattered. Existing studies have methodological limitations, small sample sizes, specific populations, and variable assessment times, making it difficult to clearly understand the true frequency of these manifestations.

In this context, we conducted a systematic review and meta-analysis of the frequency of genitourinary symptoms in patients with long COVID, aiming to provide sufficient evidence to support the proposal of a genitourinary phenotype for long COVID-19. Incorporating this phenotype into future clinical guidelines for long COVID-19 would be of great value in improving diagnostic accuracy and clinical management. It would also contribute to a better understanding of genitourinary manifestations following infection, improve the development of evidence-based follow-up strategies, and ultimately optimize patient outcomes.

## Materials and methods

This manuscript has been prepared in accordance with Preferred Reporting Items for Systematic review and Meta-Analysis (PRISMA) (Supplementary Appendix 1) [8].

### Study protocol

Registered in PROSPERO (International Prospective Register of Systematic Reviews, NIHR), ID: CRD420251082688.

### Study design

Systematic review and meta-analysis.

### Eligibility criteria

Primary studies, observational studies (cross-sectional, cohort, and case–control) evaluating individuals of any age with one or more genitourinary symptoms (such as decline in renal function, urinary incontinence, urgency, frequency, voiding dysfunction, erectile dysfunction, scrotal pain, epididymo-orchitis, or menstrual alterations) in the context of long COVID were included. Long COVID was defined as the persistence or recurrence of symptoms ≥ 12 weeks after SARS-CoV-2 infection confirmed by polymerase chain reaction (PCR), antigen testing, or clinical criteria [1, 5].

No restrictions were applied regarding language, publication year, or publication status. Studies were excluded if they included only individuals without genitourinary manifestations, those with pre-existing genitourinary disorders unrelated to COVID-19, those focused solely on the acute phase of the disease (< 12 weeks), or those that did not specify the timing of symptom onset in relation to infection. Letters to the editor, commentaries, editorials, narrative reviews, and other manuscripts without original data were also excluded.

### Information sources

The databases consulted included MEDLINE through PubMed, Scopus, Web of Science (WoS), Embase, SciELO, and Bireme-BvS, from their inception until October 2025 (the last search was conducted on October 11th, 2025). Search terms included combinations of “gynecologic disease,” “urological disorders,” “frequency”, and “Long COVID” using Boolean operators.

### Search strategies

This was carried out using the PECO framework (Population [P: Adults], Exposure [E: Long COVID], Comparator [C: not applicable], and Outcome [O: genitourinary disorders]). A structured search strategy was developed to capture the spectrum of genitourinary outcomes. The strategy combined broad condition-level terms (e.g., “gynecologic diseases” and “urological disorders”) with symptom-level and condition-specific terms, including urinary urgency, frequency, nocturia, cystitis, chronic kidney disease (CKD), decline in estimated glomerular filtration rate (eGFR), and sexual dysfunction, among other relevant descriptors. For each database, the search syntax was adapted by integrating controlled vocabulary (e.g., MeSH, DeCS, and Emtree terms) with free-text keywords, using Boolean operators and database-specific field tags where appropriate. The complete electronic search strategies for all databases, including all controlled vocabulary and free-text terms, are provided in Supplementary Appendix 2. In addition to database searches, we reviewed the reference lists of all included articles. If an article cited another study that appeared relevant based on its title, we retrieved and assessed that study according to our eligibility criteria. This process was repeated iteratively until no additional eligible studies were identified. The search strategy was externally peer reviewed by an experienced information specialist using the Peer Review of Electronic Search Strategies (PRESS) checklist to ensure accuracy, completeness, and methodological rigor. [9]

### Selection process

The documents identified from each information source were managed using Rayyan (Rayyan Systems Inc., Cambridge, MA, USA) [10]. Duplicates were removed both automatically and manually. Subsequently, titles and abstracts were screened by two pairs of reviewers (AR and DP-V), who worked independently and blinded to each other. Discrepancies between the review groups were resolved by consensus.

### Data collection and analysis

An Excel spreadsheet (PC Excel, version 15.24; 2016 Microsoft Corporation) was created for data extraction. Two authors extracted data from the included studies (AR, DP-V), and three additional authors verified the extracted data (FM, TF-L, JR). Discrepancies between reviewers were resolved by consensus.

### Variables

The variables analyzed included publication year, geographic location (country and continent), number of cases, population size, study design, COVID-19 severity, genitourinary disorder, COVID-19 diagnostic tool, and duration of long COVID. Tables provide a narrative summary of the general characteristics of all included studies. These characteristics include authors, title, journal, year of publication, region-country, design, number of participants, inclusion criteria, exclusion criteria, mean age, sex, risk of bias, and frequency. The main outcome variable was genitourinary disorders.

### Individual biases and quality

Risk of bias (RoB) for individual studies was assessed using the Joanna Briggs Institute (JBI) critical appraisal checklist for frequency studies [11–13]. This instrument evaluates nine aspects: (D1) adequacy of the sampling frame, (D2) recruitment method, (D3) sample size, (D4) study subjects and setting, (D5) coverage, (D6) diagnostic methods, (D7) reliability and standardization of measurements, (D8) statistical analysis, and (D9) response rate. Two reviewers (DP-V and AR) performed independent evaluations, marking each item as “yes,” “no,” “unclear,” or “not applicable.” Any discrepancies were discussed and resolved by consensus. The percentage of affirmative responses determined overall RoB, classifying studies as “high” risk (≤ 49%), “moderate” risk (50–69%), or “low” risk (≥ 70%). RoB summary plots across domains were generated using the ROBVIS R package v.0.3.0.900 [14].

### Synthesis method

The data from the different included studies is presented in tables. Given the relevance of the topic and the need to provide added value regarding the frequency of genitourinary alterations associated with long COVID, a quantitative synthesis is proposed using a meta-analysis with a random-effects model and a restricted maximum likelihood method. Only those genitourinary alterations with two or more studies reporting the same condition will be included in the meta-analysis. To stabilize inter-study variance (heterogeneity), the Freeman–Tukey double arcsine transformation was applied to the proportions data [15]. For clarity and clinical usefulness, the resulting estimates were back-transformed to their original proportion after the meta-analysis.

Statistical heterogeneity among the included studies was quantified using Cochran’s *Q* test, *τ*^2 ^and the *I*^2^ statistic. The *Q* test was considered statistically significant at a *p* value < 0.05. In addition, univariate meta-regression analyses were conducted to formally investigate potential sources of the observed heterogeneity, using the following prespecified covariates: sample size, RoB, duration of long COVID, COVID diagnostic tool, and COVID severity. Finally, specific subgroup sensitivity analyses are proposed to be performed in the menstrual disorders meta-analysis; a leave-one-out meta-analysis and a correction for the standard error of the proportion estimate using the Knapp-Hartung method were also conducted. All computational procedures were performed using Stata 18 software (StataCorp LLC, 2023).

### Assessment of publication bias

Publication bias was not evaluated, because fewer than ten studies were included in the analysis. According to established methodological recommendations, Egger’s regression test should not be applied when the number of studies is below this threshold, as the test has limited statistical power and may yield unreliable or spurious results under such conditions [16].

### Certainty of evidence assessment

This assessment was not performed, because no specific instrument exists to evaluate the certainty of evidence in systematic reviews of proportions.

## Results

**Fig. 1:**
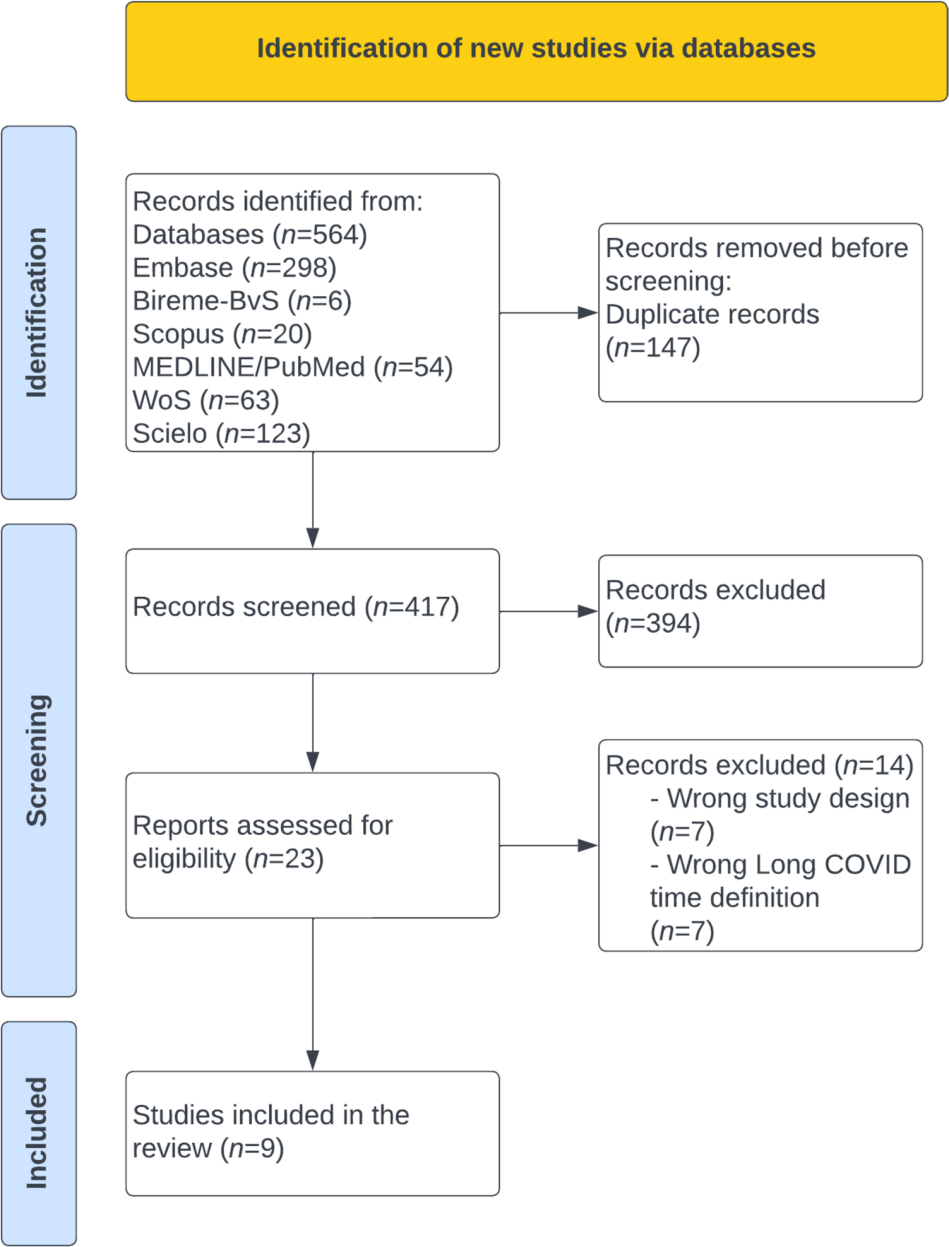
PRISMA flow diagram for studies selection 218 × 174 mm (300 × 300 DPI)

The electronic search identified 564 articles: 54 from PubMed, 6 from BVS, 20 from Scopus, 123 from SciELO, 298 from Embase, and 63 from WoS. After screening, 147 duplicates were removed. A total of 417 articles were assessed by title and abstract, resulting in 23 full-text articles evaluated for eligibility. Of these, 14 articles were excluded (7 did not meet the long COVID duration criteria, 3 letters to the editor, 4 posters). Finally, nine primary studies, including a total of 2332 participants, were included [17–25] (Fig. 1).

### Study characteristics

The included articles were published between 2021 and 2025. The studies were conducted in the following countries: Greece [17], India [18], the United States [19], China [20], Japan [21, 23], Pakistan [22], Romania [24] and Brazil [25] (Table 1).

**Table 1:** Characteristics of the included studies

Author, year	Country	Frequency, *n* (%)	Specific outcomes evaluated	Age	Females *n* (%)	Study design
Lamb, 2022[19]	USA	250 (71.4%)	Cystitis	64.5 (47–82)**	140 (40.0%)	Prospective cohort study
Zachariou, 2022 [17]	Greece	44 (66.7%)	Overactive bladder	59.5 (44–72)**	30 (45.5%)	Case–control study
Khan, 2023 [22]	Pakistan	142 (45.7%)	Menstrual disorders	34,3 ± 13,6*	311 (100%)	Cross- sectional study
		37 (25.3%)	Erectile dysfunction	32,9 ± 12,8*	0 (0%)	
Dong, 2024 [20]	China	69 (81.2%)	Menstrual disorders	32.4 ± 8.52 *	85 (100%)	Cross-sectional study
Sakurada, 2024 [21]	Japan	44 (19.7%)	Menstrual disorders	42.5 (34–48)**	223 (100%)	Retrospective cohort study
Syed, 2024 [18]	India	102 (50.0%)	Menstrual disorders	21.4 ± 1.95*	204 (100%)	Cross-sectional study
Boruga, 2024 [24]	Romania	22 (23.9%)	Decline in renal function	56.9 ± 7.6*	42 (45.7%)	Case–control study
Assisa, 2025 [25]	Brazil	83 (33.7%)	Decline in renal function	47.7 (45–50)**	155 (63.0%)	Cross-sectional study
Kato, 2025 [23]	Japan	116 (19.0%)	Erectile dysfunction	56 (48–63)**	0 (0%)	Case–control study

Age is reported as *mean ± standard deviation, ** Median (minimum and maximum values)

Overall, 51.0% of the participants were women, with ages ranging from 17 to 82 years (Table 1).

Of the included studies, 44.4% were cross-sectional [18, 20, 22, 25], 33.3% were case–control studies [17, 23, 24], 11.1% were retrospective cohort studies [21] and the remaining 11.1% were prospective cohort studies [19]. All studies (100%) were conducted in outpatient settings (Table 1).

Regarding the diagnostic method for SARS-CoV-2 infection, 55.56% of the studies used medical history [17, 20–23], 33.3% used PCR [18, 24, 25] and the remaining 11.1% relied on antigen testing [19]. With respect to the temporal criteria applied for the definition of Long COVID, 55.56% of the included studies established a 12-week duration [18, 20–22, 25]. In contrast, 11.11% defined the condition at 13 weeks [17], 11.11% at 14 weeks [19], 11.11% at 24 weeks [24], and 11.11% at 52 weeks [23]. Regarding the severity of the initial COVID-19 episode, 44.4% of the studies included patients with severe disease [17, 18, 19, 22], 11.1% addressed mild cases [21] and 44.4% moderate cases [20, 23–25] (Table 2).

**Table 2:** Severity of COVID, long COVID time, and COVID diagnostic tool

Author, year	Severity of COVID	Long COVID time	COVID diagnostic tool
Lamb, 2022 [19]	Severe	14 weeks	Antigen test
Zachariou, 2022 [17]	Severe	13 weeks	History of having contracted
Khan, 2023 [22]	Severe	12 weeks	History of having contracted
Dong, 2024 [20]	Moderate	12 weeks	History of having contracted
Sakurada, 2024 [21]	Mild	12 weeks	History of having contracted
Syed, 2024 [18]	Severe	12 weeks	PCR
Boruga, 2024 [24]	Moderate	24 weeks	PCR
Assis, 2025 [25]	Moderate	12 weeks	PCR
Kato, 2025 [23]	Moderate	52 weeks	History of having contracted

Severity of COVID was classified according to the criteria reported in each primary study. Long COVID time is expressed in weeks from acute infection to outcome assessment. COVID diagnostic tool refers to the method used to confirm prior SARS-CoV-2 infection (e.g., PCR, antigen test, or documented history of infection)

### Risk of bias

**Fig. 2:**
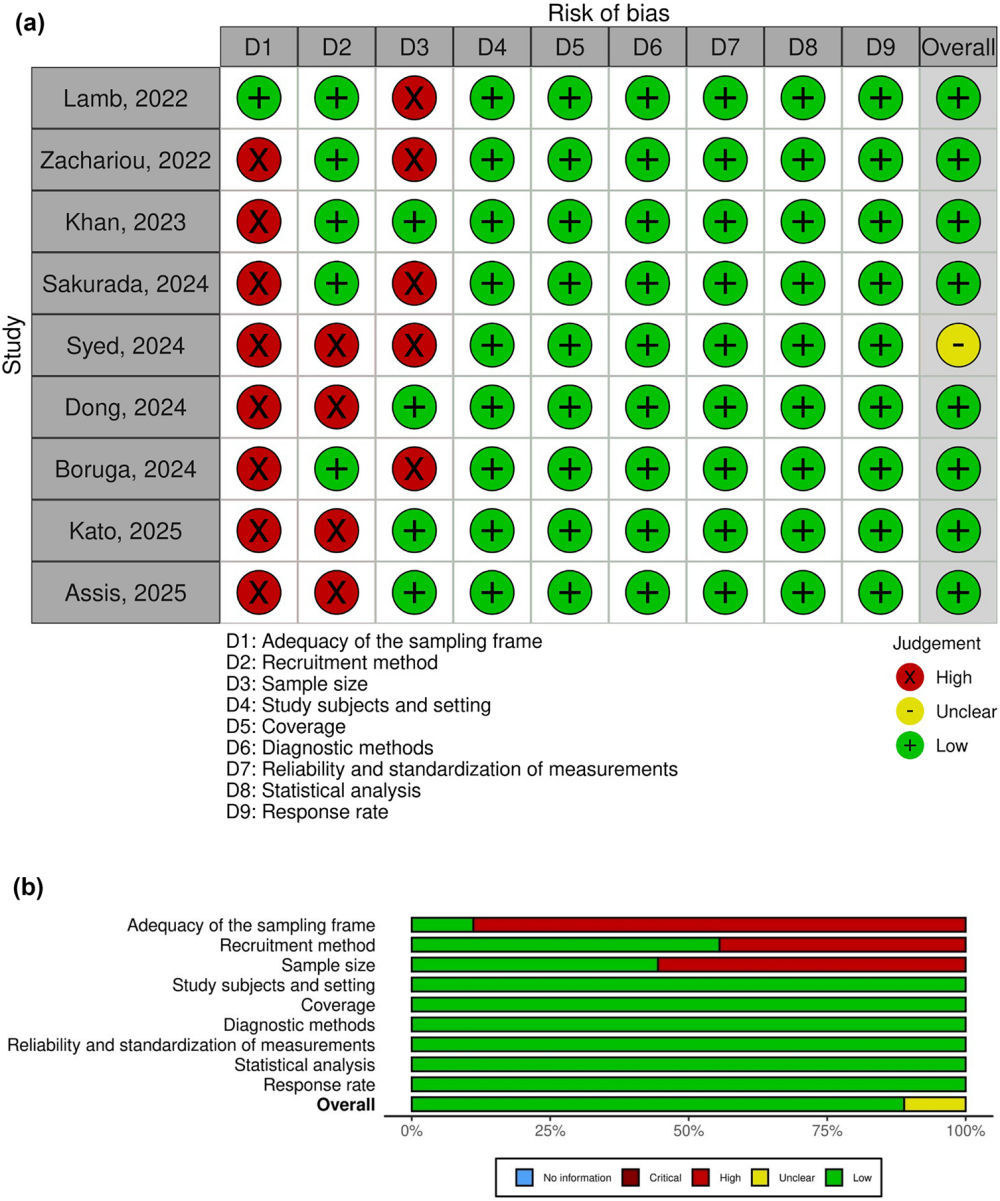
Risk of bias of the articles included. **a** Individual assessment; **b** assessment for each domain

Figure 2 shows the overall RoB assessment for the included studies. Overall, 8 (88.9%) of the studies were classified as low risk, 1 (11.1%) as moderate risk, and none as high risk.

In the domain-specific RoB assessment, although most domains were rated as low RoB, important concerns were identified regarding the adequacy of the sampling frame (D1) and recruitment methods (D2), with several studies judged to have a high risk. These limitations were mainly related to non-probabilistic sampling strategies and insufficient reporting of participant recruitment procedures. In addition, deficiencies were observed in the sample size domain (D3), largely due to the absence of sample size justification. In contrast, most studies reported low risk across the domains of diagnostic method, statistical analysis, coverage, and measurement reliability, reinforcing the methodological robustness of the included evidence. The distribution of RoB assessments across domains is presented in Fig. 2.

### Genitourinary symptoms frequency

The data from the included studies are presented in Tables 1 and 2. Table 1 primarily reports the frequency of genitourinary symptoms across the included studies, along with the general study characteristics described in the Study Characteristics section. Table 2 provides additional clinical information, including the severity of acute COVID-19, the symptoms duration used to define long COVID, and the diagnostic method used to confirm prior SARS-CoV-2 infection.

### Menstrual disorders

**Fig. 3:**
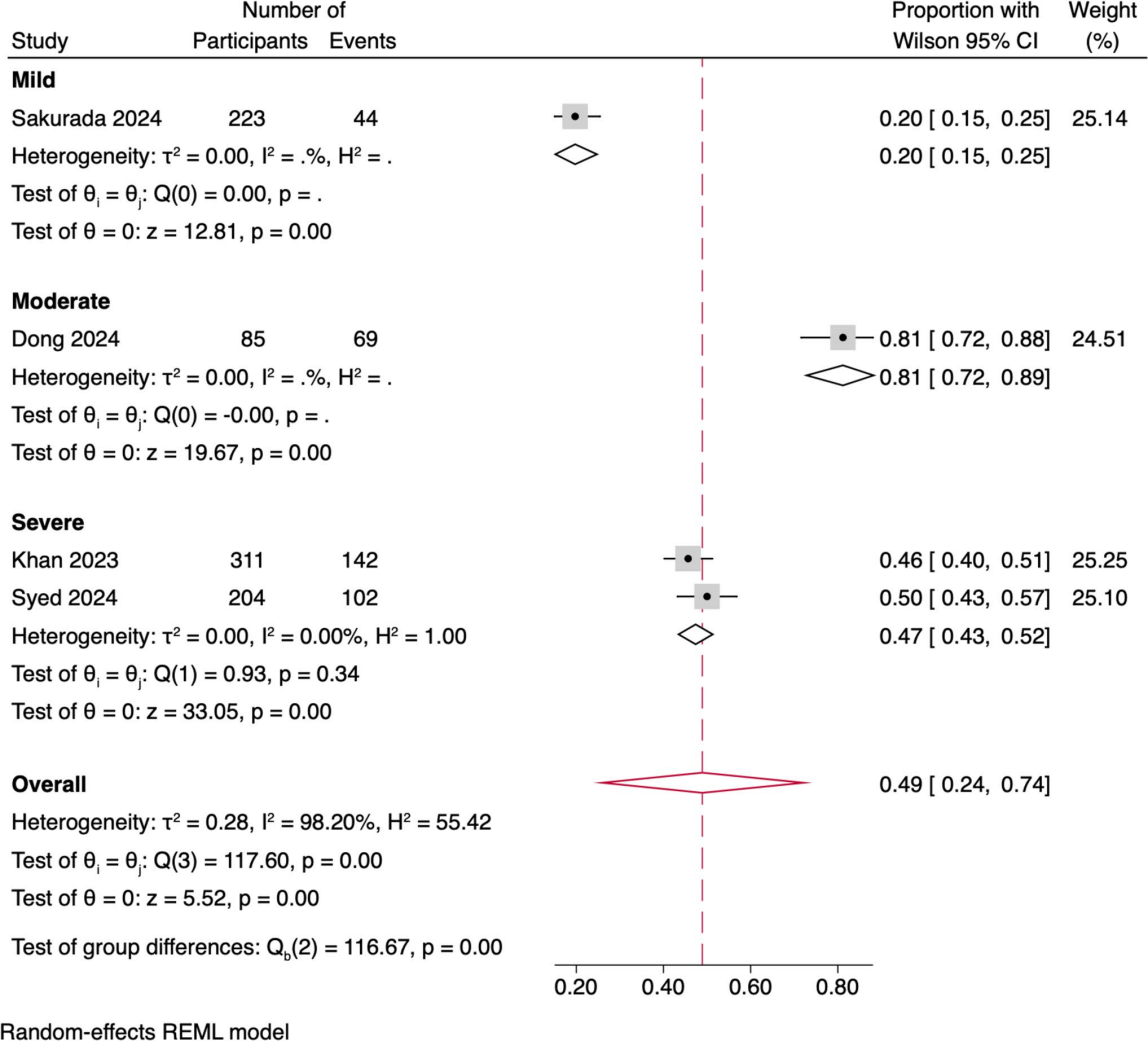
Forest plot of the frequency of menstrual disorders in long COVID patients by COVID-19 severity

Of the nine articles, four were related to menstrual disorders in patients with long COVID. The total number of patients included across these 4 studies was 823 women. These disorders were subclassified into premenstrual syndrome, dysmenorrhea, and abnormal menstruation syndrome (Table 3). An overall frequency of menstrual disorders in patients with long COVID of 49% (95% CI 24–74) was observed (Fig. 3).

**Table 3:** Menstrual disorders

Author, year	Population (*n*)	Premenstrual syndrome *n* (%)	Dysmenorrhea *n* (%)	Abnormal menstrual syndrome *n* (%)
Khan, 2023 [22]	311	59 (19.0%)	32 (10.3%)	57 (18.3%)
Dong, 2024 [20]	69	47 (68.1%)	28 (40.6%)	56 (81.2%)
Sakurada, 2024 [21^24^]	44	7 (15.9%)	11 (25.0%)	28 (63.6%)
Syed, 2024 [18]	102	59 (57.8%)	46 (45.1%)	19 (18.6%)

Values are presented as number and percentage (*n*, %), calculated using the total female population of each study as the denominator. Population (*n*) corresponds to the total number of female participants included in the analysis of menstrual outcomes

When stratified by clinical severity, 20% (95% CI 15–25) in mild cases, 81% (95% CI 72–89) in moderate cases, and 47% (95% CI 43–52) in severe cases were observed. The comparison between groups showed statistically significant differences (Qb = 116.67, *p* < 0.001), indicating that the frequency of detected cases varies significantly according to clinical severity, being higher in patients with moderate forms of the disease (Fig. 3).

In the analysis based on the COVID diagnostic method, the pooled proportion in the clinical history subgroup was 49% (95% CI 15–83), with very high heterogeneity (*I*^2^ = 98.74%, *τ*^2^ = 0.42, *p* < 0.001), while in the PCR subgroup, the proportion was 50% (95% CI 43–57). No statistically significant differences were observed between the subgroups (Qb = 0.00, *p* = 0.94), indicating that the proportion of identified cases was similar regardless of the diagnostic method used. (Supplementary Appendix 3).

By addressing the RoB, the frequency of menstrual disorders was 49% (95% CI 15–83) in studies with low risk and 50% (95% CI 43–57) in studies with moderate risk. No significant differences were found between the groups. However, heterogeneity was substantial among the low-risk studies (*I*^2^ = 98.74%, *τ*^2^ = 0.42, *p* < 0.001), suggesting variability in study design or sample characteristics (Supplementary Appendix 4).

The leave-one-out meta-analysis demonstrated the robustness of the pooled estimate for the proportion of menstrual disorders. The sequential exclusion of each individual study did not significantly alter the overall pooled estimate. Although the proportions varied slightly, ranging from 38 to 59% after the omission of a study, all resulting estimations remained consistent and proximal to the general outcome (Supplementary Appendix 5). This finding confirms that the combined estimate is not excessively influenced by any single study, thereby reinforcing the reliability of the meta-analysis results. In addition, with the Knapp–Hartung correction for the estimation, the frequency of menstrual disorders was 49% (95% CI 11–87).

### Erectile dysfunction

**Fig. 4:**
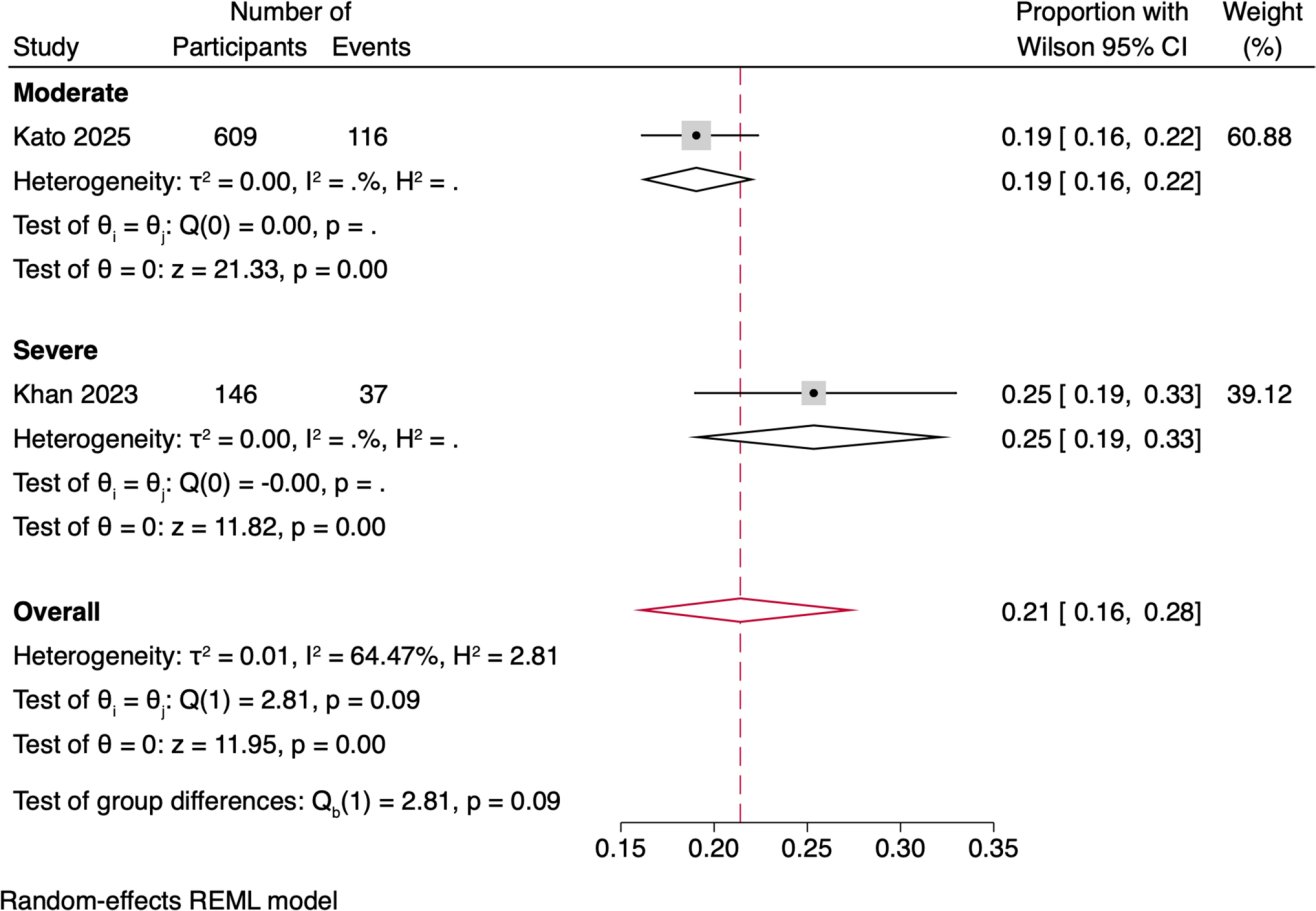
Forest plot of the frequency of erectile dysfunction in long COVID patients by COVID-19 severity

Of the 9 articles, 2 focused on erectile dysfunction among patients with long COVID. The total number of patients included across these 2 studies was 755. A frequency of 21% (95% CI 16–28) was observed among long COVID patients, with moderate heterogeneity (*I*^2^ = 64.47%, *τ*^2^ = 0.01, *p* = 0.09) (Fig. 4).

In the analysis of subgroups by COVID-19 severity, the proportion of moderate cases was 19% (95% CI 16–22), while the proportion of severe cases was 25% (95% CI 19–33). No statistically significant differences were found between the two groups (Qb = 2.81, *p* = 0.09), suggesting that symptom frequency was comparable across clinical severity levels (Fig. 4).

### Renal function decline

**Fig. 5:**
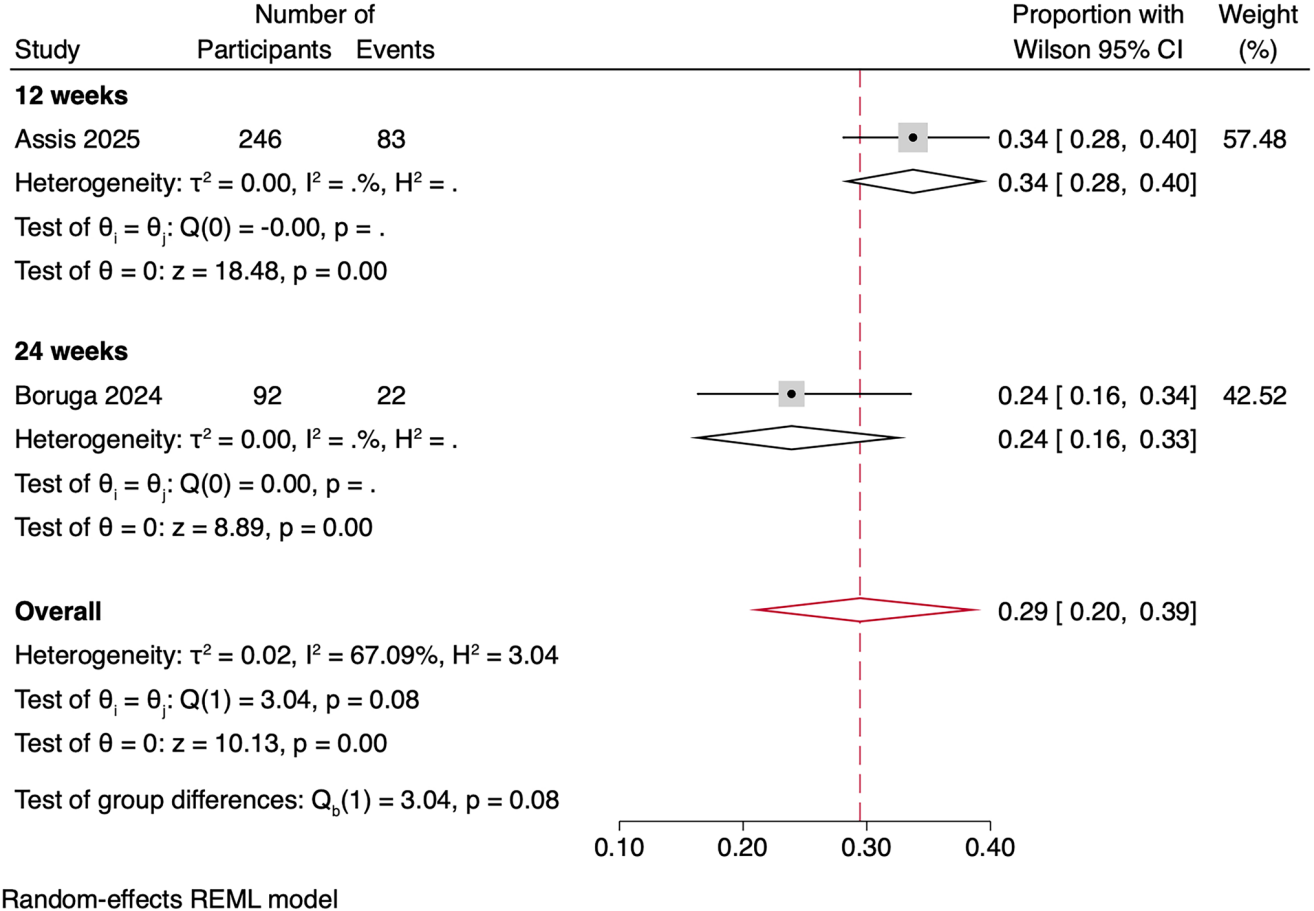
Forest plot of the frequency of decrease in renal function in long COVID patients by the duration of long COVID

Out of 9 articles, 2 examined renal decline in long COVID patients, involving a total of 338 individuals. The frequency of this condition among long COVID patients was found to be 29% (95% CI 20–39), with moderate heterogeneity (*I*^2^ = 67.09%, *τ*^2^ = 0.02, *p* = 0.08) (Fig. 5). When stratified by follow-up duration, the proportion was 34% (95% CI 28–40) in the 12-week subgroup, and 24% (95% CI 16–34) in the 24-week subgroup. The comparison between groups based on follow-up duration did not show statistically significant differences (Qb = 3.04, *p* = 0.08), which suggests that the frequency of detected renal disorders was comparable between patients followed up at 12 weeks and those followed up at 24 weeks (Fig. 5).

### Cystitis and overactive bladder

Among the 9 articles included in the analysis, one focused on the prevalence of cystitis in patients with long COVID. A total of 350 patients were included in this study, of which 250 (71.4%) were diagnosed with cystitis. Of the 250 patients with cystitis, 140 were females (40.0%). The patients’ ages ranged from 47 to 82 years, with a mean of 64.5 ± 7.0 years. These findings suggest a high frequency of cystitis in this population; however, the lack of additional studies limited the ability to conduct a meta-analysis. The full results are presented in Tables 1 and 2.

Regarding overactive bladder, another study included a total of 66 patients, of whom 44 (66.7%) were diagnosed with this condition. All 44 patients were females (100%). The patients’ ages ranged from 44 to 72 years, with a mean of 59.5 ± 4.0 years. As there is only one study on this pathology in patients with long COVID, a meta-analysis could not be performed, and the results are presented individually. Detailed results are available in Tables 1 and 2.

### Source of heterogeneity

The univariate meta-regression analyses were non-significant for the sample size, RoB, duration of long COVID, and COVID diagnostic tool covariates. However, when assessing the severity of COVID, the heterogeneity appears to be explained by this covariate (R^2^: 100; p < 0.001; I^2^ for residual heterogeneity: 0%).

## Discussion

Long COVID has been described as a complex syndrome affecting multiple organs and systems of the human body, with the hematologic and nervous systems being the most studied. However, the genitourinary system, due to its essential role in homeostasis, reproductive function, and quality of life (QoL), represents an area of particular interest. Although reports indicate the presence of genitourinary alterations in patients with long COVID, most correspond to primary studies with small sample sizes, which limits the strength of the evidence [26, 27]. In this study, we observed a frequency of 49% for menstrual disturbances, 21% for erectile dysfunction, and 29% for renal decline among patients with long COVID.

The pathophysiology underlying these manifestations is likely multifactorial and involves several systemic mechanisms described in the literature. In women with long COVID, reports have documented increased menstrual volume, intermenstrual bleeding, heavier flow, and prolonged cycles, accompanied by elevated serum levels of inflammatory cytokines (such as TNF-α and IL-8) and alterations in androgen receptor expression within the endometrium. These findings suggest that SARS-CoV-2 infection may disrupt the hypothalamic-pituitary–gonadal axis, induce endometrial or vascular dysfunction, and consequently lead to menstrual irregularities [28].

In this study, most COVID-19 diagnoses were made based on medical history, reflecting the reality of a recent disease that caused a global pandemic, during which diagnostic resources were limited, laboratory tests were not widely available, and protocols were still under development [29]. Despite the variability in the diagnostic method, the frequency of long COVID symptoms was found to be almost similar between patients diagnosed based on medical history and those confirmed by PCR, indicating that the diagnostic method did not significantly influence the identification of patients with persistent symptoms. This is supported by previous studies, which show that although RT-PCR remains the gold standard, medical history can be a valid tool for identifying COVID-19 cases, especially in resource-limited settings or during the early phases of the pandemic [29].

Several studies have shown that the severity of the initial COVID-19 illness is clearly associated with a higher risk of developing long COVID, indicating that patients who required hospitalization, supplemental oxygen, or experienced a greater number of symptoms during the acute phase are more likely to continue exhibiting long-term clinical manifestations [30–32]. In this regard, this study reinforces this association, showing that in cases with mild infection, the frequency of menstrual disturbances was low, whereas it increased considerably in those with severe disease. Although a higher frequency was observed in the moderate severity group, this result should be interpreted as a finding limited by the scarce available evidence, as it comes from a single study with a small number of participants.

Regarding COVID-19 severity, it is important to note that in the present study, the heterogeneity observed in menstrual alterations was attributable to the severity subgroup. This finding suggests that disease severity plays a key role in the variability of menstrual disturbances among patients with long COVID, potentially reflecting differential systemic inflammatory responses or hormonal dysregulation across severity levels.

This finding warrants further analysis, as menstrual function is predominantly hormonal and is highly sensitive to various internal and external factors. Among these factors, environmental ones stand out, particularly psychological stress, which has been associated with menstrual irregularities, premenstrual syndrome, and dysmenorrhea in different contexts and populations [33–35]. For example, AlShehri et al. (2018) found that 91% of female university students experienced some menstrual problem related to high stress [27]; studies in Ethiopia reported a frequency of menstrual irregularity of 32.6%, with a significant association with stress and sleep deprivation [28], while research during the COVID-19 pandemic also showed increases in menstrual alterations related to psychological stress [29]. Therefore, the menstrual alterations observed in patients with long COVID could be influenced not only by the direct effects of the virus but also by environmental and psychological factors inherent to the pandemic context, which justifies further studies to elucidate these mechanisms.

Furthermore, some studies were conducted in student populations, which may constrain generalizability to older or more heterogeneous groups. However, this subgroup represented 204 of 2,332 participants (8.7%) and did not contribute disproportionate weight to the meta-analysis or demonstrate effect sizes meaningfully different from the overall estimate. While demographic composition should be considered when extrapolating these findings, this subgroup is unlikely to have materially influenced the overall conclusions.

In addition to biological and environmental considerations, the statistical properties of the pooled estimates warrant careful attention. The pooled prevalence of menstrual disorders (49%, 95% CI 24–74) was accompanied by substantial between-study heterogeneity, limiting its applicability as a uniform summary measure across settings. Accordingly, these pooled proportions should be regarded as a synthesis of diverse study-specific findings rather than precise population-level estimates.

Regarding erectile dysfunction, evidence indicates that long COVID can impair endothelial function, penile microcirculation, and promote chronic pro-inflammatory or dysautonomic states, thereby compromising erectile capacity [36].

Despite the marked association between COVID-19 severity and the frequency of menstrual disturbances, this variable was found to have limited or no impact on erectile dysfunction. This disparity suggests that menstrual alterations may be more directly linked to disruption of the hormonal axis and the intense systemic inflammatory response accompanying more severe forms of acute disease. In contrast, long-term erectile dysfunction appears to be primarily driven by chronic vascular mechanisms (such as endothelial dysfunction or microvascular damage), whose ultimate manifestation is less dependent on the initial clinical severity. Nevertheless, this interpretation should be approached with caution, as the meta-analysis on erectile dysfunction is based on a considerably smaller number of studies compared with the menstrual disturbances group, which reduces the statistical power to detect significant intergroup differences and warrants further investigation.

Renal decline, although less consistently reported, is thought to arise from persistent viral infection, microvascular injury, renin–angiotensin system dysregulation, and chronic low-grade inflammation leading to progressive renal function decline.

A prospective study conducted in South Korea demonstrated that as time passes from the acute phase of COVID-19 infection, there is an increased risk of developing long COVID [37]. In this study, at 6-month post-infection, 59.8% of patients had at least one persistent symptom, and at 24 months, this increased to 71.2% [25]. In contrast to these findings, our results for renal decline showed a different temporal trend: the frequency of renal disorders at 12 weeks was higher than at 24 weeks. This pattern is likely due to the kidney being an organ directly targeted by the virus during the acute phase of the infection. While long-term vascular damage may contribute to persistent renal decline, the initial high frequency appears to reduce as the acute inflammation subsides. This suggests that the pathophysiology of long COVID renal disorders has a stronger component related to the resolution of acute viral and inflammatory injury, setting it apart from the progressive nature observed in conditions, such as menstrual disturbances.

While some studies have reported renal damage as a consequence of COVID-19, including acute tubular necrosis, mitochondrial impairment, and tubulointerstitial damage, these factors can significantly affect renal decline. Moreover, the presence of viral nucleocapsid protein, macrophage infiltration, and immune-mediated mechanisms further supports the hypothesis of renal damage induced by COVID-19 [38]. However, it is important to recognize that several confounding factors may influence renal outcomes in long COVID patients, including baseline kidney function, acute kidney injury during the initial infection, and comorbid conditions, such as hypertension, diabetes, and nephrotoxic exposures. Given the limited data available and the presence of these confounders, it is crucial to emphasize that causality cannot be inferred from the current studies. Therefore, further research is needed to better understand the relationship between long COVID and renal function decline, accounting for these underlying conditions.

Finally, we also identified lower urinary tract alterations, including cystitis and overactive bladder; however, only one study was available for each condition, limiting their overall impact. Reports of long COVID bladder dysfunction describe new-onset urgency, frequency, and nocturia, potentially mediated by inflammatory cytokine-induced urothelial injury or ACE2 receptor expression in bladder mucosa [39].

Genitourinary alterations observed in long COVID are not merely clinical findings but have important implications for patients’ QoL. Persistent urinary symptoms such as urgency, frequency, nocturia, and incontinence have been associated with significant discomfort and social embarrassment, often leading to sleep disturbances, activity limitations, and psychological distress, thereby reducing overall QoL. For example, new or worsening overactive bladder symptoms in long COVID have been linked to higher symptom and QoL burden scores, reflecting the daily impact of these conditions on patients’ lives [19]. Sexual dysfunction, including erectile dysfunction and other forms of sexual impairment, has also been reported as a common manifestation of long COVID and may further contribute to emotional distress, relationship difficulties, and reduced self-esteem [20]. Renal function decline, while often subclinical, can be associated with long-term decreases in estimated glomerular filtration rate and increased risk of chronic kidney disease, which may influence physical functioning and necessitate ongoing medical follow-up [24]. Taken together, genitourinary symptoms in long COVID can exert a multidimensional impact on physical, psychological, and social aspects, highlighting the importance of comprehensive evaluation and management to address QoL in this population.

Emerging evidence suggests that long COVID can present as a candidate genitourinary phenotype, characterized by renal dysfunction, menstrual irregularities, and erectile impairment. These manifestations are not isolated organ-specific complications, but rather may reflect a shared pathobiological axis involving ACE2 dysregulation, endothelial injury, microvascular inflammation, and neuroendocrine imbalance. The genitourinary phenotype of long COVID may represent a microvascular–endocrine syndrome, where ACE2 downregulation, endothelial damage, persistent inflammation, and hormonal dysregulation converge to produce renal, menstrual, and sexual dysfunction [38, 40, 41]. This possible emergent phenotype expands our understanding of long COVID by highlighting the involvement of the genitourinary system, an area that has been relatively underexplored compared to other sequelae, such as respiratory, neurological, and cardiovascular manifestations.

While these genitourinary features share common characteristics, it is crucial to emphasize that certain confounders, such as comorbid conditions and baseline organ function, must still be considered. As such, it remains unclear whether the proposed emergent genitourinary phenotype could serve as a clinical screening construct for follow-up and referral, and further research is needed to determine its potential as a diagnostic tool in long COVID.

### Limitations

During the course of this study, certain limitations were encountered, primarily related to the availability of research that strictly met the inclusion criteria. This is largely due to the fact that long COVID is a relatively recent clinical entity, which limits the number of studies with adequate methodological design and sufficient follow-up time. Many publications describing genitourinary manifestations after SARS-CoV-2 infection were excluded, because they did not clearly define a post-acute period longer than 12 weeks, a fundamental criterion for classifying cases as long COVID.

Furthermore, studies addressing genitourinary symptoms or alterations potentially associated with COVID-19 vaccination were not included, as the objective of this review was to analyze manifestations directly related to the pathophysiological mechanisms of SARS-CoV-2 infection. While several publications have described possible associations between the virus and genitourinary alterations—such as scrotal discomfort, cystitis, or epididymo-orchitis—most referred to the acute or subacute phases and, therefore, did not meet the temporal definition required for long COVID.

In addition, erectile dysfunction and renal function decline are each supported by only two studies, which limits the strength of the conclusions that can be drawn from these findings. Given this, it is important to acknowledge these outcomes with caution, as more research with larger, independent data sets is needed to validate their relevance and further investigate their role within long COVID.

Finally, further research is strongly recommended to better define the frequency, mechanisms, and long-term clinical impact of genitourinary manifestations within the long COVID spectrum.

## Conclusions

Our findings demonstrate a significant frequency of genitourinary alterations, particularly menstrual irregularities, among individuals with long COVID, underscoring the relevance of this system in the spectrum of long COVID sequelae. While the strength of the available evidence is limited by small sample sizes and methodological heterogeneity, our results suggest that the severity of the initial infection plays an important role in the development of these symptoms. Recognizing these manifestations is essential for improving diagnostic accuracy and guiding comprehensive management strategies, as well as for informing future research aimed at elucidating the underlying mechanisms and long-term impact of long COVID on genitourinary health.

## Supplementary Information

Below is the link to the electronic supplementary material.

Supplementary file1 PRISMA checklist (DOCX 16 KB)

Supplementary file2 Complete search strategy (JPG 200 KB)

Supplementary file3 Forest plot of the pooled frequency of menstrual disorders according to the COVID-19 diagnostic method used (JPG 499 KB)

Supplementary file4 Forest plot of the pooled frequency of menstrual disorders by RoB (JPG 496 KB)

Supplementary file5 Leave-one-out meta-analysis for the frequency of menstrual disorders (JPG 442 KB)

## Data Availability

No data sets were generated or analyzed during the current study.
