# Supplementary material for: Unveiling the genitourinary phenotype of long COVID: a systematic review and meta-analysis
Source: Int Urol Nephrol. 2026 Mar 8;58(8):2849–62. doi: 10.1007/s11255-026-05073-9 (PMC13375746; doi:10.1007/s11255-026-05073-9)
Supplement: Supplementary file 1 — Supplementary file1 PRISMA checklist (DOCX 16 KB) [file 11255_2026_5073_MOESM1_ESM.docx]

***Supplementary Appendix 1***

| **Date** | **17 September 2025** |
| --- | --- |
| **MEDLINE through PubMed** | 1. (("Preoperative Exercise"[Majr]) AND "Cardiorespiratory Fitness"[Mesh]) AND "Surgical Procedures, Operative"[Mesh]) 2. (Exercise, Preoperative[all fields] OR Preoperative Exercises[all fields] OR Preoperative Conditioning[all fields] OR Conditioning, Preoperative[all fields] OR Preoperative Conditionings[all fields] OR Pre-operative Exercise[all fields] OR Exercise, Pre-operative[all fields] OR Pre operative Exercise[all fields] OR Pre-operative Exercises[all fields] OR Pre-operative Rehabilitation[all fields] OR Pre operative Rehabilitation[all fields] OR Pre-operative Rehabilitations[all fields] OR Rehabilitation, Pre-operative[all fields] OR Preoperative Rehabilitation[all fields] OR Preoperative Rehabilitations[all fields] OR Rehabilitation, Preoperative[all fields] OR Pre-operative Conditioning[all fields] OR Conditioning, Pre-operative[all fields] OR Pre operative Conditioning[all fields] OR Pre-operative Conditionings[all fields] OR Prehabilitation[all fields]) 3. **("Cardiorespiratory Fitness"[all fields] OR "Cardiopulmonary Fitness"[all fields] OR "Cardiovascular Fitness"[all fields] OR "Aerobic Capacity"[all fields] OR "Aerobic Fitness"[all fields] OR "Cardiorespiratory Endurance"[all fields] OR "Cardiovascular Endurance"[all fields] OR "Maximal Oxygen Uptake"[all fields] OR "VO2max"[all fields] OR "VO2 max"[all fields] OR "VO2 peak"[all fields] OR "Peak Oxygen Consumption"[all fields] OR "Oxygen Uptake"[all fields] OR "Oxygen Consumption"[all fields] OR "Exercise Capacity"[all fields] OR "Physical Fitness"[all fields])** 4. **("major surgery"[all fields] OR "major surgical procedure"[all fields] OR "major operation"[all fields] OR "major surgical intervention"[all fields] OR "complex surgery"[all fields] OR "extensive surgery"[all fields] OR "invasive surgery"[all fields] OR "elective surgery"[all fields] OR "high-risk surgery"[all fields] OR "high complexity surgery"[all fields] OR "traditional surgery"[all fields] OR "open surgery"[all fields] OR "non-minimally invasive surgery"[all fields] OR "major abdominal surgery"[all fields] OR "major thoracic surgery"[all fields] OR "major orthopedic surgery"[all fields] OR "major cardiovascular surgery"[all fields] OR "major oncologic surgery"[all fields] OR "major cancer surgery"[all fields] OR "curative surgery"[all fields] OR "radical surgery"[all fields] OR "advanced surgery"[all fields] OR "multidisciplinary surgery"[all fields] OR "long-duration surgery"[all fields] OR "major elective surgery"[all fields] OR "planned major surgery"[all fields] OR "scheduled major surgery"[all fields])** 5. ((#2) AND (#3)) AND (#4)) |
| **Embase** | 1. 'preoperative exercise'/exp AND 'cardiorespiratory fitness'/exp AND 'surgical procedure'/exp 2. 'exercise, preoperative' OR 'preoperative exercises' OR 'preoperative conditioning' OR 'conditioning, preoperative' OR 'preoperative conditionings' OR 'pre-operative exercise' OR 'exercise, pre-operative' OR 'pre operative exercise' OR 'pre-operative exercises' OR 'pre-operative rehabilitation' OR 'pre operative rehabilitation' OR 'pre-operative rehabilitations' OR 'rehabilitation, pre-operative' OR 'preoperative rehabilitation' OR 'preoperative rehabilitations' OR 'rehabilitation, preoperative' OR 'pre-operative conditioning' OR 'conditioning, pre-operative' OR 'pre operative conditioning' OR 'pre-operative conditionings' OR 'prehabilitation' 3. 'Cardiorespiratory Fitness' OR 'Cardiopulmonary Fitness' OR 'Cardiovascular Fitness' OR 'Aerobic Capacity' OR 'Aerobic Fitness' OR 'Cardiorespiratory Endurance' OR 'Cardiovascular Endurance' OR 'Maximal Oxygen Uptake' OR 'VO2max' OR 'VO2 max' OR 'VO2 peak' OR 'Peak Oxygen Consumption' OR 'Oxygen Uptake' OR 'Oxygen Consumption' OR 'Exercise Capacity' OR 'Physical Fitness' 4. 'major surgery' OR 'major surgical procedure' OR 'major operation' OR 'major surgical intervention' OR 'complex surgery' OR 'extensive surgery' OR 'invasive surgery' OR 'elective surgery' OR 'high-risk surgery' OR 'high complexity surgery' OR 'traditional surgery' OR 'open surgery' OR 'non-minimally invasive surgery' OR 'major abdominal surgery' OR 'major thoracic surgery' OR 'major orthopedic surgery' OR 'major cardiovascular surgery' OR 'major oncologic surgery' OR 'major cancer surgery' OR 'curative surgery' OR 'radical surgery' OR 'advanced surgery' OR 'multidisciplinary surgery' OR 'long-duration surgery' OR 'major elective surgery' OR 'planned major surgery' OR 'scheduled major surgery' 5. #2 AND #3 AND #4 |
| **Web of Science** | 1. TS=("Exercise, Preoperative" OR "Preoperative Exercises" OR "Preoperative Conditioning" OR "Conditioning, Preoperative" OR "Preoperative Conditionings" OR "Pre-operative Exercise" OR "Exercise, Pre-operative" OR "Pre operative Exercise" OR "Pre-operative Exercises" OR "Pre-operative Rehabilitation" OR "Pre operative Rehabilitation" OR "Pre-operative Rehabilitations" OR "Rehabilitation, Pre-operative" OR "Preoperative Rehabilitation" OR "Preoperative Rehabilitations" OR "Rehabilitation, Preoperative" OR "Pre-operative Conditioning" OR "Conditioning, Pre-operative" OR "Pre operative Conditioning" OR "Pre-operative Conditionings" OR "Prehabilitation") 2. TS=("Cardiorespiratory Fitness" OR "Cardiopulmonary Fitness" OR "Cardiovascular Fitness" OR "Aerobic Capacity" OR "Aerobic Fitness" OR "Cardiorespiratory Endurance" OR "Cardiovascular Endurance" OR "Maximal Oxygen Uptake" OR "VO2max" OR "VO2 max" OR "VO2 peak" OR "Peak Oxygen Consumption" OR "Oxygen Uptake" OR "Oxygen Consumption" OR "Exercise Capacity" OR "Physical Fitness") 3. TS=("major surgery" OR "major surgical procedure" OR "major operation" OR "major surgical intervention" OR "complex surgery" OR "extensive surgery" OR "invasive surgery" OR "elective surgery" OR "high-risk surgery" OR "high complexity surgery" OR "traditional surgery" OR "open surgery" OR "non-minimally invasive surgery" OR "major abdominal surgery" OR "major thoracic surgery" OR "major orthopedic surgery" OR "major cardiovascular surgery" OR "major oncologic surgery" OR "major cancer surgery" OR "curative surgery" OR "radical surgery" OR "advanced surgery" OR "multidisciplinary surgery" OR "long-duration surgery" OR "major elective surgery" OR "planned major surgery" OR "scheduled major surgery") 4. #1 AND #2 AND #3 |
| **Scopus** | 1. TITLE-ABS("Exercise, Preoperative" OR "Preoperative Exercises" OR "Preoperative Conditioning" OR "Conditioning, Preoperative" OR "Preoperative Conditionings" OR "Pre-operative Exercise" OR "Exercise, Pre-operative" OR "Pre operative Exercise" OR "Pre-operative Exercises" OR "Pre-operative Rehabilitation" OR "Pre operative Rehabilitation" OR "Pre-operative Rehabilitations" OR "Rehabilitation, Pre-operative" OR "Preoperative Rehabilitation" OR "Preoperative Rehabilitations" OR "Rehabilitation, Preoperative" OR "Pre-operative Conditioning" OR "Conditioning, Pre-operative" OR "Pre operative Conditioning" OR "Pre-operative Conditionings" OR "Prehabilitation") 2. TITLE-ABS("Cardiorespiratory Fitness" OR "Cardiopulmonary Fitness" OR "Cardiovascular Fitness" OR "Aerobic Capacity" OR "Aerobic Fitness" OR "Cardiorespiratory Endurance" OR "Cardiovascular Endurance" OR "Maximal Oxygen Uptake" OR "VO2max" OR "VO2 max" OR "VO2 peak" OR "Peak Oxygen Consumption" OR "Oxygen Uptake" OR "Oxygen Consumption" OR "Exercise Capacity" OR "Physical Fitness") 3. TITLE-ABS("major surgery" OR "major surgical procedure" OR "major operation" OR "major surgical intervention" OR "complex surgery" OR "extensive surgery" OR "invasive surgery" OR "elective surgery" OR "high-risk surgery" OR "high complexity surgery" OR "traditional surgery" OR "open surgery" OR "non-minimally invasive surgery" OR "major abdominal surgery" OR "major thoracic surgery" OR "major orthopedic surgery" OR "major cardiovascular surgery" OR "major oncologic surgery" OR "major cancer surgery" OR "curative surgery" OR "radical surgery" OR "advanced surgery" OR "multidisciplinary surgery" OR "long-duration surgery" OR "major elective surgery" OR "planned major surgery" OR "scheduled major surgery") 4. #1 AND #2 AND #3 |
| **SCIELO** | (prehabilitación OR prehabilitacion OR prehabilitation OR "ejercicio preoperatorio" OR "exercício pré-operatório" OR "rehabilitación preoperatoria" OR "reabilitação pré-operatória") AND ("aptitud física" OR "aptidão física" OR fitness OR "capacidad aeróbica" OR "capacidade aeróbica" OR VO2max OR "consumo de oxígeno" OR "consumo de oxigênio") AND ("cirugía mayor" OR "cirurgia de grande porte" OR "cirugía compleja" OR "cirurgia complexa" OR "cirugía oncológica" OR "cirurgia oncológica" OR "cirugía abdominal" OR "cirurgia abdominal") |
| Biblioteca virtual de salud | (prehabilitación OR prehabilitacion OR prehabilitation OR "ejercicio preoperatorio" OR "exercício pré-operatório" OR "rehabilitación preoperatoria" OR "reabilitação pré-operatória") AND ("aptitud física" OR "aptidão física" OR fitness OR "capacidad aeróbica" OR "capacidade aeróbica" OR VO2max OR "consumo de oxígeno" OR "consumo de oxigênio") AND ("cirugía mayor" OR "cirurgia de grande porte" OR "cirugía compleja" OR "cirurgia complexa" OR "cirugía oncológica" OR "cirurgia oncológica" OR "cirugía abdominal" OR "cirurgia abdominal") |
