# Supplementary figures and images for: Unveiling the genitourinary phenotype of long COVID: a systematic review and meta-analysis
Source: Int Urol Nephrol. 2026 Mar 8;58(8):2849–62. doi: 10.1007/s11255-026-05073-9 (PMC13375746; doi:10.1007/s11255-026-05073-9)

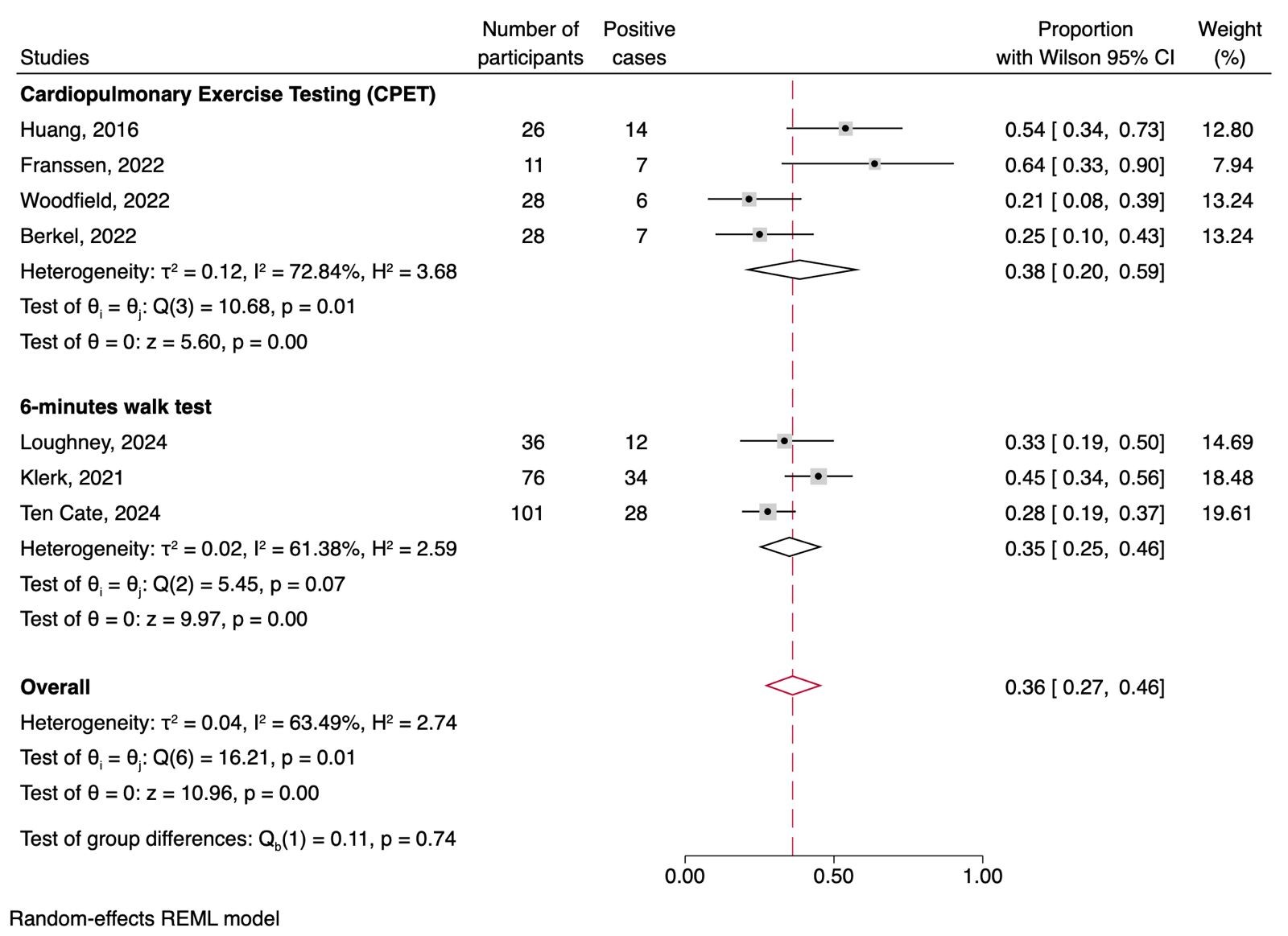

Supplement: Supplementary file 2 — Supplementary file2 Complete search strategy (JPG 200 KB) [file 11255_2026_5073_MOESM2_ESM.jpg]

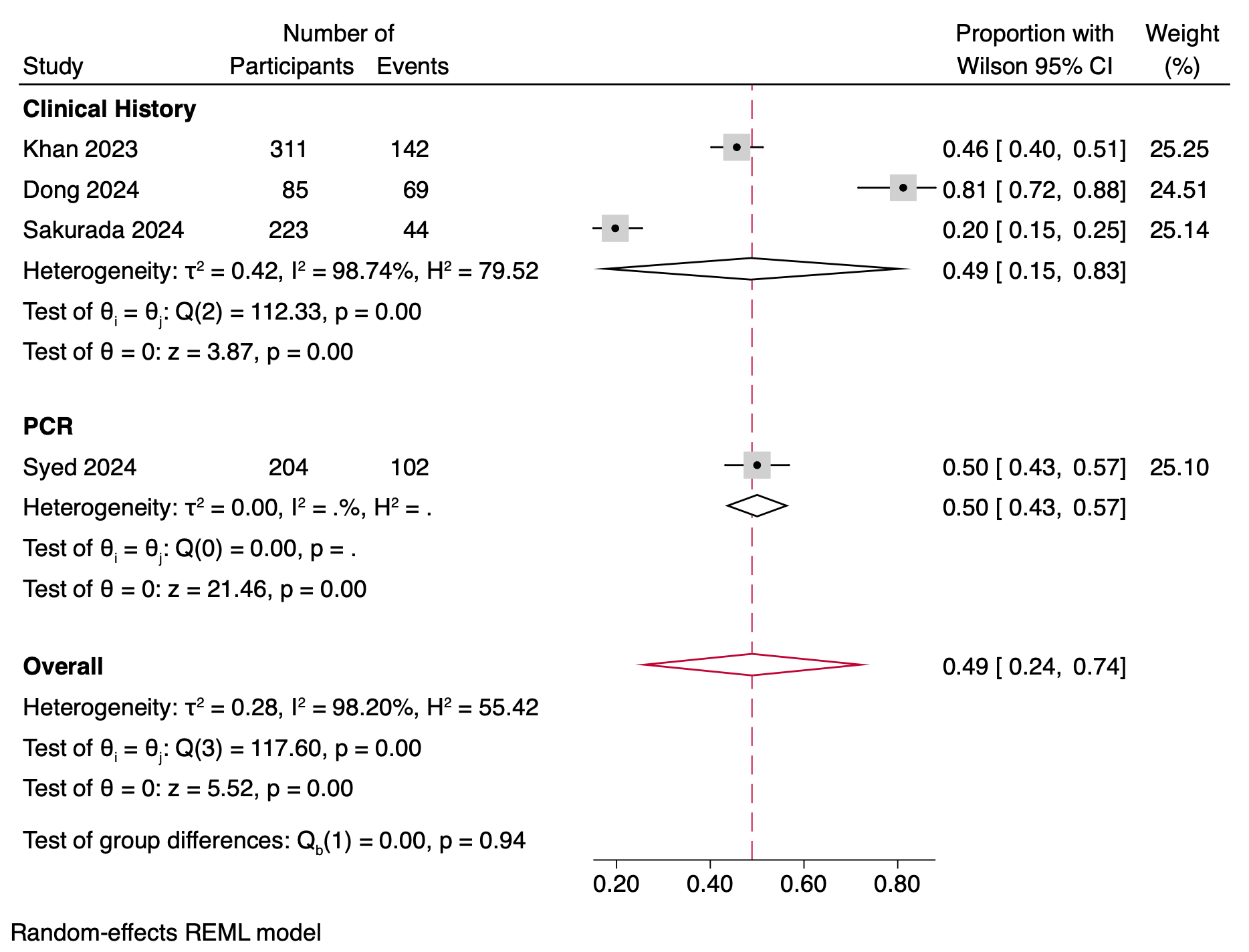

Supplement: Supplementary file 3 — Supplementary file3 Forest plot of the pooled frequency of menstrual disorders according to the COVID-19 diagnostic method used (JPG 499 KB) [file 11255_2026_5073_MOESM3_ESM.jpg]

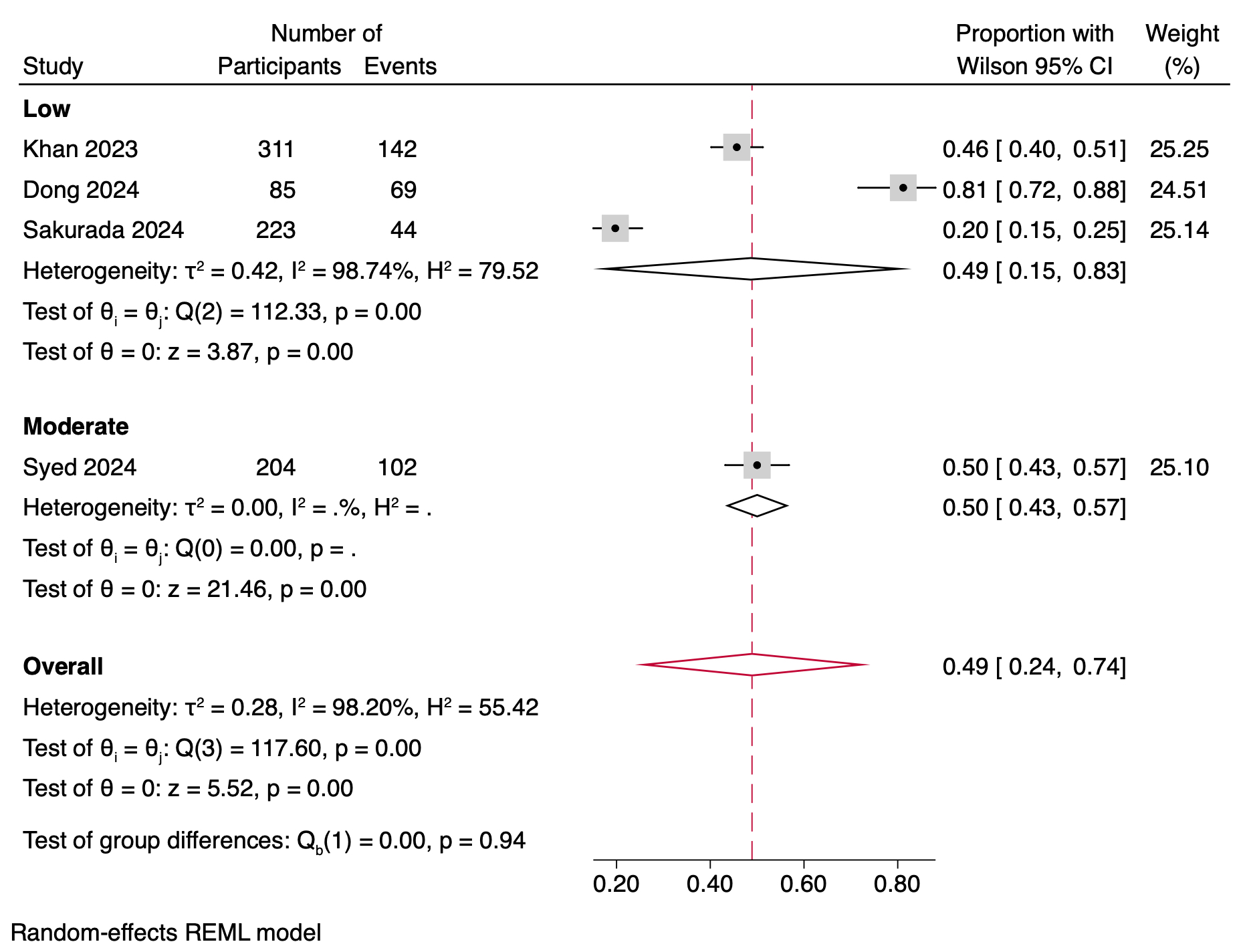

Supplement: Supplementary file 4 — Supplementary file4 Forest plot of the pooled frequency of menstrual disorders by RoB (JPG 496 KB) [file 11255_2026_5073_MOESM4_ESM.jpg]

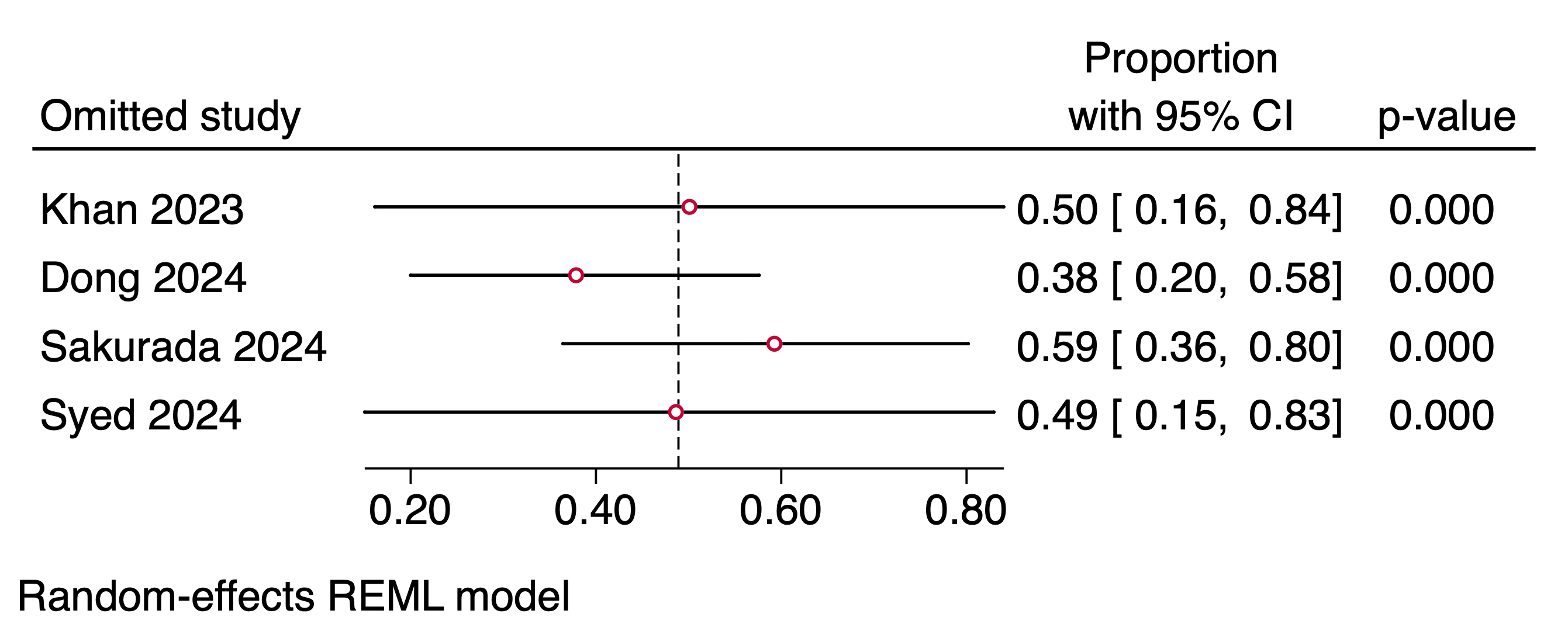

Supplement: Supplementary file 5 — Supplementary file5 Leave-one-out meta-analysis for the frequency of menstrual disorders (JPG 442 KB) [file 11255_2026_5073_MOESM5_ESM.jpg]
